# The complete mitochondrial genome of *Annamanum lunulatum* (Coleoptera: Lamiinae) and its phylogeny

**DOI:** 10.1080/23802359.2019.1710284

**Published:** 2020-01-14

**Authors:** Xin-Yi Dai, Huan Zhang, Xiao-Dong Xu, Yi-Yang Jia, Jia-Yong Zhang, Dan-Na Yu, Hong-Yi Cheng

**Affiliations:** a College of Chemistry and Life Science, Zhejiang Normal University, Jinhua, China;; b Key Lab of Wildlife Biotechnology, Conservation and Utilization of Zhejiang Province, Zhejiang Normal University, Jinhua, China

**Keywords:** Longhorn beetle, Lamiinae, mitogenome, phylogenetic relationship

## Abstract

The complete mitochondrial genome of the *Annamanum lunulatum* is 15,610 bp in length, which contains 13 protein-coding genes, 22 transfer RNAs, two ribosomal RNAs, and the A + T-rich region. The arrangement of genes is identical to all know longhorn beetles mitochondrial genomes. The overall AT content of the mitochondrial genome is 75.3%, whereas the AT content of A + T-rich region is 84.3%. In ML and BI phylogenetic analyses, *A. lunulatum* is a sister clade to *Blepephaeus succinctor*, and the monophyly of Lamiinae is supported.

Coleoptera (Hexapoda: Insecta) are a group of insects with over 360,000 described species of beetles (Hunt et al. 2007). It is in the larval stage that some species of Coleoptera belonging to pests make strong damage to plants. *Annamanum lunulatum* (Cerambycidae: Lamiinae) is a longhorn beetle firstly reported by Pic (1934) and *Uraecha longzhouensis* is considered as one of its junior synonyms (Lin and Lingafelter 2018). The genus *Annamanum* includes 31 described species located in South China, Japan, Vietnam, Laos, Cambodia, India, Myanmar, and Malaysia, among which 15 species were reported from China (Holzschuh 2017; Lin and Ge 2017; Tavakilian and Chevillotte 2018; Yang and Yang 2019). However, there were few genetic information on the mitochondrial genome reported in this genus. Hence, we sequenced the mitochondrial genome of *A. lunulatum* to benefit the studies of the genetic diversity in *Annamanum* and to discuss its phylogenetic relationship within Lamiinae.

Samples of *A. lunulatum* were collected from Jinxiu (22°57′28″ N, 107°11′38″ E), Guangxi province, China on 10 August 2016 and classified by JY Zhang. The sample (JX20160810) was identified and stored at 40 °C in the Animal Specimen Museum, College of Life Sciences and Chemistry, Zhejiang Normal University, China. The total genomic DNA was extracted from leg muscle tissue using an Ezup Column Animal Genomic DNA Purification Kit (Sangon Biotech Company, Shanghai, China) and stored in the Zhang’s laboratory. Eight universal primers and six specific primers (Simon et al. 2006; Zhang et al. 2008; Zhang, Cai, et al. 2018; Zhang, Yu, et al. 2018) were utilized for polymerase chain reaction (PCR) amplification. The mitochondrial genome was deposited in GenBank with an accession number MN356095.

The complete mitochondrial genome of *A. lunulatum* is 15,610 bp in length, including 13 protein-coding genes, 22 transfer RNAs, two ribosomal RNAs, and the A + T-rich region. The overall AT content of the mitochondrial genome is 75.3%, whereas the AT content of A + T-rich region is 84.3%. Almost all the protein-coding genes use ATN as an initiation codon excluding *ND1* with TTG. Eleven genes (*ND1*, *ND2*, *ND3*, *ND4L*, *COI*, *COII*, *COIII*, *ATP6*, *ATP8*, *ND6*, *Cyt b*) end the typical stop codon TAA or TAG, whereas *ND4* and *ND5* end with T—.

In order to reconstruct the phylogenetic relationships of Lamiinae, we used 13 protein-coding genes of 24 species, which contained *Galeruca daurica* as the outgroup (Kim et al. 2009; Lu et al. 2011; Chiu et al. 2016; Fang et al. 2016; Guo et al. 2016; Li et al. 2016; Wang et al. 2016; Lim et al. 2017; Liu et al. 2017; Yang et al. 2017; Liu et al. 2018; Que et al. 2019; Wang, Dai, et al. 2019; Wang, Lan, et al. 2019). MrBayes version 3.2 and the RAxML version 8 programs (Ronquist et al. 2012; Stamatakis 2014) were used to construct BI and ML trees, respectively (Figure 1). *Annamanum lunulatum* was a sister clade to *Blepephaeus*, and the monophyly of Lamiinae was supported in both BI and ML analyses.

**Figure 1.**
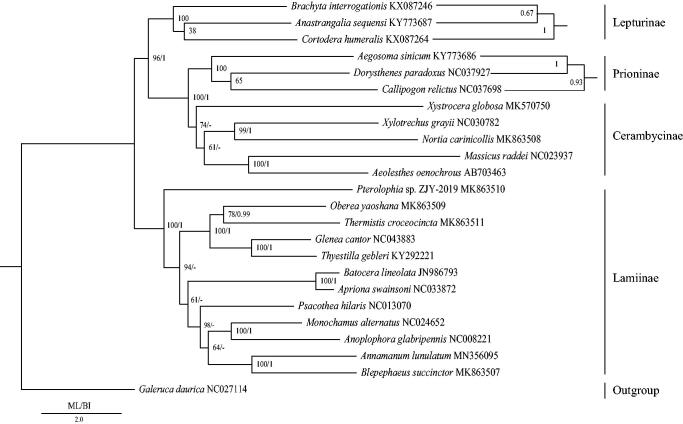
Phylogenetic trees of the relationships among 24 species of Coleoptera, including *Annamanum lunulatum*, were based on the nucleotide dataset of the 13 mitochondrial protein-coding genes. The numbers showed between branches indicate the posteriori probabilities from Bayesian inference (BI) and bootstrap percentages from maximum-likelihood (ML, 1000 replications) analyses. The GenBank accession numbers of all species were also shown.

## References

[CIT0001] Chiu WC, Yeh WB, Chen ME, Yang MM. 2016. Complete mitochondrial genome of *Aeolesthes oenochrous* (Fairmaire) (Coleoptera: Cerambycidae): an endangered and colorful longhorn beetle. Mitochondrial DNA A. 27(1):686–687. 10.3109/19401736.2014.913143 24810074

[CIT0002] Fang J, Qian L, Xu M, Yang XJ, Wang BD, An YL. 2016. The complete nucleotide sequence of the mitochondrial genome of the Asian longhorn beetle, *Anoplophora glabripennis* (Coleoptera: Cerambycidae). Mitochondrial DNA A. 27(5):3299–3300. 10.3109/19401736.2015.1015012 25693709

[CIT0003] Guo K, Chen J, Xu CQ, Qiao HL, Xu R, Zhao XJ. 2016. The complete mitochondrial genome of the longhorn beetle *Xylotrechus grayii* (Coleoptera: Cerambycidae). Mitochondrial DNA A. 27:2133–2134. 10.3109/19401736.2014.982592 25423529

[CIT0004] Holzschuh C. 2017. Neue Arten von Bockkäfern aus der Tribus Clytini und der Unterfamilie Lamiinae (Coleoptera, Cerambycidae) vom asiatischen Festland. Acta Musei Moraviae. Scientiae Biologicae (Brno). 102:93–138.

[CIT0005] Hunt T, Bergsten J, Levkanicova Z, Papadopoulou A, John OS, Wild R, Hammond PM, Ahrens D, Balke M, Caterino MS, et al. 2007. A comprehensive phylogeny of beetles reveals the evolutionary origins of a superradiation. Science. 318(5858):1913–1916. 18096805 10.1126/science.1146954

[CIT0006] Kim KG, Hong MY, Kim MJ, Im HH, Kim MI, Bae CH, Seo SJ, Lee SH, Kim I. 2009. Complete mitochondrial genome sequence of the yellow-spotted long-horned beetle *Psacothea hilaris* (Coleoptera: Cerambycidae) and phylogenetic analysis among coleopteran insects. Mol Cells. 27(4):429–441. 19390824 10.1007/s10059-009-0064-5

[CIT0007] Li F, Zhang H, Wang W, Weng H, Meng Z. 2016. Complete mitochondrial genome of the Japanese pine sawyer, *Monochamus alternatus* (Coleoptera: Cerambycidae). Mitochondrial DNA A. 27(2):1144–1145. 10.3109/19401736.2014.936321 24989053

[CIT0008] Lim J, Yi DK, Kim Y H, Lee W, Kim S, Kang JH, Kim IK. 2017. Complete mitochondrial genome of *Callipogon relictus* Semenov (Coleoptera: Cerambycidae): a natural monument and endangered species in Korea. Mitochondrial DNA B. 2(2):629–631. 10.1080/23802359.2017.1372718 PMC7800709 33473925

[CIT0009] Lin MY, Lingafelter SW. 2018. Taxonomic notes on Chinese Lamiini (Coleoptera: Cerambycidae: Lamiinae). Zootaxa. 4482(2):367–374. 30313826 10.11646/zootaxa.4482.2.8

[CIT0010] Lin MY, Ge SQ. 2017. Notes on the genera *Annamanum* Pic and *Uraecha* Thomson (Coleoptera: Cerambycidae: Lamiinae: Lamiini). Humanity Space Int Almanac. 6:889–915.

[CIT0011] Liu JH, Jia PF, Luo T, Wang QM. 2017. Complete mitochondrial genome of white-striped long-horned beetle, *Batocera lineolata* (Coleoptera: Cerambycidae) by next-generation sequencing and its phylogenetic relationship within superfamily Chrysomeloidea. Mitochondrial DNA B. 2(2):520–521. 10.1080/23802359.2017.1361797 PMC7800789 33473883

[CIT0012] Liu YQ, Chen DB, Liu HH, Hu HL, Bian HX, Zhang RS, Yang RS, Jiang XF, Shi SL. 2018. The complete mitochondrial genome of the longhorn beetle Dorysthenes Paradoxus (Coleoptera: Cerambycidae: Prionini) and the implication for the phylogenetic relationships of the Cerambycidae species. J Insect Sci. 18(2):21:1–8. 10.1093/jisesa/iey012 PMC5833319 29718483

[CIT0013] Lu W, Wang Q, Tian MY, Xu J, Qin AZ. 2011. Phenology and laboratory rearing procedures of an Asian longicorn beetle, *Glenea cantor* (Coleoptera: Cerambycidae: Lamiinae). J Econ Entomol. 104(2):509–516. 21510199 10.1603/ec10345

[CIT0014] Pic M. 1934. Nouveautés asiatiques. Matériaux Pour Servir à L’étude Des Longicornes. 11:33–40.

[CIT0015] Que S, Yu A, Liu P, Jin M, Xie GA. 2019. The complete mitochondrial genome of *Apriona swainsoni*. Mitochondrial DNA B. 4(1):931–932.

[CIT0016] Ronquist F, Teslenko M, Mark PVD, Ayres DL, Darling A, Höhna S, Larget B, Liu L, Suchard MA, Huelsenbeck JP. 2012. MrBayes 3.2: efficient Bayesian phylogenetic inference and model choice across a large model space. Syst Biol. 61(3):539–542. 22357727 10.1093/sysbio/sys029 PMC3329765

[CIT0017] Simon C, Buckley TR, Frati F, Stewart JB, Beckenbach AT. 2006. Incorporating molecular evolution into phylogenetic analysis, and a new compilation of conserved polymerase chain reaction primers for animal mitochondrial DNA. Annu Rev Ecol Evol Syst. 37(1):545–579.

[CIT0018] Stamatakis A. 2014. RAxML version 8: a tool for phylogenetic analysis and post-analysis of large phylogenies. Bioinformatics. 30(9):1312–1313. 24451623 10.1093/bioinformatics/btu033 PMC3998144

[CIT0019] Tavakilian G, Chevillotte H. 2018. Titan: base de données internationales sur les Cerambycidae ou Longicornes. Version 4.0. [accessed June 2019]. http://titan.gbif.fr/index.html.

[CIT0020] Wang J, Dai XY, Xu XD, Zhang ZY, Yu DN, Storey KB, Zhang JY. 2019. The complete mitochondrial genomes of five longicorn beetles (Coleoptera: Cerambycidae) and phylogenetic relationships within Cerambycidae. PeerJ. 7:e7633. 31534857 10.7717/peerj.7633 PMC6732212

[CIT0021] Wang J, Lan DY, Dai XY, Yu DN, Storey KB, Zhang JY. 2019. The complete mitochondrial genome of *Xystrocera globose* (Coleoptera: Cerambycidae) and its phylogeny. Mitochondrial DNA B. 4(1):1647–1649.

[CIT0022] Wang YT, Liu YX, Tong XL, Ren QP, Jiang GF. 2016. The complete mitochondrial genome of the longhorn beetle, *Massicus raddei*. Mitochondrial DNA A. 27(1):209–211. 10.3109/19401736.2014.880892 24491106

[CIT0023] Yang J, Cao LJ, Geng YX, Wei DF, Chen M. 2017. Determination of the complete mitochondrial genome of *Thyestilla gebleri* and comparative analysis of the mitochondrial genome in Cerambycidae. Chinese J Appl Entomol. 54:755–766.

[CIT0024] Yang SL, Yang SY. 2019. *Annamanum flavimaculatum*, a new species of longhorn beetle (Coleoptera, Cerambycidae) from China. Zookeys. 889:49–56. 31777436 10.3897/zookeys.889.38296 PMC6872599

[CIT0025] Zhang JY, Zhou CF, Gai YH, Song DX, Zhou KY. 2008. The complete mitochondrial genome of *Parafronurus youi* (Insecta: Ephemeroptera) and phylogenetic position of the Ephemeroptera. Gene. 424(1-2):18–24. 18725275 10.1016/j.gene.2008.07.037

[CIT0026] Zhang LP, Cai YY, Yu DN, Storey KB, Zhang JY. 2018. Gene characteristics of the complete mitochondrial genomes of *Paratoxodera polyacantha* and *Toxodera hauseri* (Mantodea: Toxoderidae). PeerJ. 6:e459. 10.7717/peerj.4595 PMC5911385 29686943

[CIT0027] Zhang LP, Yu DN, Storey K B, Cheng HY, Zhang JY. 2018. Higher tRNA gene duplication in mitogenomes of praying mantises (Dictyoptera, Mantodea) and the phylogeny within Mantodea. Int J Biol Macromol. 111:787–795. 29307803 10.1016/j.ijbiomac.2018.01.016

